# Very Delayed Acute Hepatitis after Pembrolizumab Therapy for Advanced Malignancy: How Long Should We Watch?

**DOI:** 10.3390/curroncol28010088

**Published:** 2021-02-14

**Authors:** Timothy Phan, Kurvi Patwala, Lara Lipton, Virginia Knight, Ahmad Aga, Stephen Pianko

**Affiliations:** 1 Monash Health, Clayton, Melbourne, VIC 3168, Australia; kurvi.patwala@monashhealth.org (K.P.); spianko@geds.com.au (S.P.); 2 The Royal Melbourne Hospital, Parkville, VIC 3052, Australia; Lara.Lipton@mh.org.au; 3 Cabrini Medical Centre, Malvern, VIC 3144, Australia; Virginia.knight@monash.edu (V.K.); ahmad.aga@mps.com.au (A.A.); 4 School of Clinical Sciences, Monash University, Melbourne, VIC 3168, Australia

**Keywords:** autoimmune hepatitis, pembrolizumab, immune checkpoint inhibitor, immune-related adverse events, neoplasms

## Abstract

Immune checkpoint inhibitors (ICIs) have led to major therapeutic advances in the management of malignancy. Despite promising outcomes for some cancers, ICIs are linked to unique side-effects known as immune-related adverse events (IrAEs). These may affect a wide array of organ systems. In particular, ICI-induced hepatitis is diagnostically challenging given its variable natural history and clinical manifestations. The onset of ICI-induced hepatitis often occurs between 6 and 14 weeks after treatment initiation and rarely exhibits delayed presentations or manifests after treatment cessation. We present a case of very delayed-onset ICI-induced hepatitis, stressing the importance of long-term surveillance for immune-indued hepatitis in patients initiated on ICIs even long after treatment cessation.

## 1. Introduction

Immune checkpoint inhibitors (ICIs) have markedly improved the prognosis of patients with some cancers. These novel agents augment the immune system by downregulating inhibitors of the anti-cancer immune response, including: cytotoxic T-lymphocyte-associated antigen 4 (CTLA4), program cell death receptor 1 (PD-1) and its ligand—programmed cell death ligand 1 (PD-L1) [1]. Despite promising clinical outcomes, ICIs are linked to unique side-effects known as immune-related adverse events (IrAEs) due to the induction of autoimmunity. IrAEs have the potential to affect a wide range of organ systems, commonly including: dermatological (skin rashes), endocrine, gastrointestinal and hepatic. As ICI therapy becomes more widespread in cancer management, IrAEs are becoming more common, including immune-induced hepatitis. We present a case of very delayed-onset ICI-induced hepatitis that occurred 7 months post-treatment cessation, thereby highlighting the importance of prolonged monitoring for an acute hepatitis with careful consideration of its diagnostic and treatment nuances. 

## 2. Case Report

Written informed consent has been obtained from the patient to publish this paper.

A 78-year-old female with stage IIIC breast cancer presented with subacute onset of jaundice. Her breast cancer had been treated with pembrolizumab for 5 months and ceased 7 months prior to presentation. She developed a prodrome of progressive fatigue and anorexia over 2 weeks. There were no recent medication changes other than oral mesalazine, which was commenced 3 months prior due to a new diagnosis of unspecified left-sided colitis confirmed during colonoscopy (possibly immune mediated). 

She was diagnosed with stage IIIB triple negative inflammatory breast cancer in 2015. This was treated with neoadjuvant chemotherapy and surgery with subsequent adjuvant radiotherapy. Approximately 12 months prior to her presentation, she developed a locally advanced right breast recurrence requiring neoadjuvant chemotherapy and PD-1 inhibitor (pembrolizumab) prior to double mastectomy. 

Initial biochemistry revealed a severe transaminitis. Her bilirubin was 199 μmol/L (<20 μmol/L) with an alanine transferase (ALT) of 1519 U/L (<40 U/L), gamma-glutamyltransferase (GGT) of 440 U/L (<60U/L) and alkaline phosphatase (ALP) of 208 (<130 U/L). She had preserved synthetic function with an international normalised ratio (INR) of 1.0 (<1.2) and an albumin of 31 g/L (34–54 g/L). Her viral hepatitis serology including hepatitis E virus, cytomegalovirus and Epstein–Barr virus was negative. A liver autoantibody screen was non-contributory. Her immunoglobulin G (IgG) titre was 9.4g/L (7–16 g/L). A liver ultrasound and subsequent magnetic resonance cholangiopancreatography (MRCP) showed no structural abnormalities or metastatic disease. 

**Figure 1:**
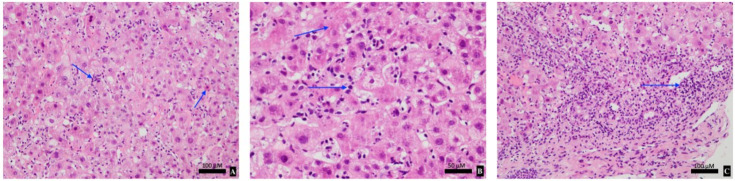
Histologic examination of liver lobule with hematoxylin and Eosin (H&E) staining demonstrating features of immune checkpoint inhibitor (ICI)-induced hepatitis with moderate lobular hepatitis, scattered apoptic hepatocytes (**A**), necro-inflammatory foci with lymphocytosis (**B**) and portal inflammation with interface activity (**C**).

**Figure 2:**
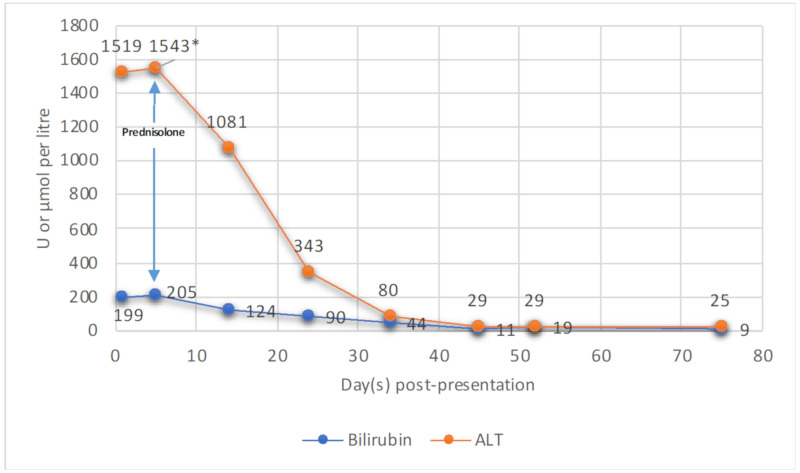
Graphical representation of bilirubin (μmol/L) and alanine transferase (ALT [U/L]) trends over a 75-day follow-up period from time of presentation. Prednisolone was commenced on Day 5 post-presentation (*).

The patient was admitted for 2 weeks with a provisional diagnosis of drug-induced liver injury (DILI) secondary to mesalazine or immunotherapy. Mesalazine was ceased and she was managed expectantly. On Day 5, her liver function tests (LFTs) worsened, her bilirubin peaked at 205 μmol/L with an ALT of 1543 U/L. A liver biopsy revealed pan-lobular hepatitis with scattered apoptic hepatocytes, necro-inflammatory foci, lymphocytosis and foci of interface activity consistent with ICI-induced hepatitis (Figure 1). Given the biopsy results, she was initiated on 50 mg of oral prednisolone (1 mg/kg/day) to minimise glucocorticoid-related side effects. The patient’s clinical and biochemical status improved with a daily downtrend in LFTs, and resolution of her fatigue and anorexia (Figure 2). She was subsequently discharged with regular LFT monitoring and a slow glucocorticoid taper. 

Considering her biopsy results and rapid resolution of liver function whilst on glucocorticoid therapy, a diagnosis of delayed immune-induced hepatitis secondary to pembrolizumab was made. 

## 3. Discussion

The clinical manifestations and natural history of ICI-induced hepatitis are heterogenous. Acute hepatitis occurs in 2–10% of patients on ICI therapy, which typically presents as an asymptomatic transaminitis [2,3]. Occasionally, patients may develop a rapidly progressive acute hepatitis and even fulminant liver failure associated with significant morbidity and mortality [4,5]. Contrary to the usual timeframe for disease onset, our patient developed ICI-induced hepatitis 7 months post-treatment cessation. Only one other author has reported a case of delayed ICI-induced hepatitis occurring 8 months post-nivolumab cessation [6]. Delayed-onset immune-induced hepatitis after treatment cessation is rare and most reported cases occur during active treatment—between 6 and 14 weeks after treatment initiation [7]. Hence, this case serves as a reminder to strictly monitor liver function monitoring up to 12 months post-treatment cessation to detect an evolving hepatitis. 

ICI-induced hepatitis can be pathologically difficult to differentiate from autoimmune hepatitis (AIH) or drug-induced liver injury (DILI). Contrasting with AIH, auto-antibodies including anti-nuclear and anti-smooth muscle antibodies may be negative in ICI-induced hepatitis. Similarly, serum IgG levels are normal or slightly elevated in ICI-induced hepatitis as exemplified by our case [2]. Liver biopsy is often not required in suspected ICI-induced hepatitis and is only indicated to exclude other causes of acute hepatitis or quantify the degree of hepatocellular injury. Biopsy findings in ICI-induced hepatitis are variable. Most commonly, panlobular hepatitis is observed in approximately 70% of cases, typified by scattered focal necrosis and acidophilic bodies [8]. Histopathological factors which differentiate AIH and DILI from ICP-hepatitis are summarised in Table 1 [2]. In our case, liver biopsy demonstrated classical features of ICI-induced hepatitis without features of AIH or DILI from mesalazine. The cellular infiltrate was suggestive of ICI-related pathology given a lymphocytic predominance without plasmacytosis nor eosinophilia. Detailed knowledge around the pathological and biochemical intricacies of ICI-induced hepatitis can greatly assist with prompt diagnosis and treatment.

Management of ICI-induced hepatitis is based on the Common Terminology Criteria for Adverse Events (CTCAE), which grades severity of disease from 1–4 [3]. Our patient presented with grade 4 hepatitis which was glucocorticoid responsive. Current guidelines advocate for prompt glucocorticoid treatment with methylprednisolone or equivalent at 1–2 mg/kg/day for three days (up to 1 g/day), followed by prednisolone 1–2 mg/kg/day over 4 weeks. In steroid-refractory cases, the introduction of mycophenolate or azathioprine is prudent. Permanent discontinuation of ICI therapy is recommended in grade 3–4 hepatitis [3,9]. Corticosteroid therapy in ICI-induced hepatitis is contentious. Spontaneous improvement in LFTs can occur with observation and low doses of corticosteroids may be sufficient to control disease [5]. Our patient was promptly commenced on prednisolone therapy at a relatively low starting dose compared with pulsed methylprednisolone with rapid clinical response and excellent efficacy. Our case supports that LFT resolution is achievable with a lower dose glucocorticoid, thereby reducing glucocorticoid-related side effects. Further high-quality randomised-controlled studies are required to assess optimal management strategies for ICI-induced hepatitis. 

Delayed-onset IrAEs of any organ system are underrecognised and clinical vigilance is critical even after treatment cessation [10]. The median interval to delayed-onset IrAE diagnosis is 6 months post-immunotherapy cessation (IQR 3–28 months) [10]. Currently, there is a paucity of evidence on delayed-onset IrAEs due to limited follow up periods and incompleteness of IrAE reporting in immuno-oncology clinical trials [11]. Best practice guidelines allude to monitoring for delayed-onset IrAEs up to 12 months after treatment discontinuation [9]. However, late-onset IrAEs are difficult to predict with available investigations and are challenging to prevent. Surveillance for IrAEs should be individualised based on a patient’s risk profile for developing IrAEs. A patient’s risk for IrAEs is increased in the presence of patient and treatment factors including: pre-existing connective tissue, vasculitic or auto-immune disease, and combination ICI usage [1]. The utility of active surveillance strategies for delayed-onset IrAEs remains an area of active research with no evidence-based algorithms available. 

## 4. Conclusions

ICIs are becoming an integral part of cancer therapy, and monitoring for adverse events requires awareness from all physicians and not just oncologists. This case pertinently emphasises the importance of long-term surveillance for immune-induced hepatitis in patients initiated on ICI therapy. Attentive appraisal of symptoms, signs and investigations is required for prompt diagnosis of ICI-induced hepatitis allowing for the timely introduction of therapy. Systemic treatment is nuanced and further high-quality evidence is required to guide optimal therapy. We advocate for individualised surveillance strategies for delayed-onset IrAEs up to 12-months after treatment cessation. However, stringent evidence-based surveillance protocols remains an area of ongoing development. 

## 5. Clinical Practice Points

- Delayed-onset ICI-induced hepatitis can occur up to 12 months post-treatment cessation;
- Autoimmune biomarkers are often negative in ICI-induced hepatitis and plasmacytosis or eosinophilia is uncommon in the biopsy cellular infiltrate;
- Low-dose glucocorticoid therapy may be warranted in select ICI-induced hepatitis patients, but further randomized control studies are required to assess optimal glucocorticoid dosing;
- Routine monitoring for delayed-onset IrAEs including liver function should be individualised and can extend up to 12 months post-treatment cessation.

## Figures and Tables

**Table 1:** Comparison of histopathological features of ICI-induced hepatitis, autoimmune hepatitis (AIH) and drug-induced liver injury (DILI). Adapted from Zen et al [2].

Histological Features	ICI-Hepatitis	AIH	DILI
**Confluent Necrosis**	Less common	More common	More common
**Eosinophilic infiltration**	Uncommon	Not specific	More common
**Bile plugs**	Uncommon	Not specific	More common
**Plasmacytosis**	Uncommon	Common	Not specific
**Hepatocellular rosettes**	Uncommon	Common	Not specific
**Emperipolesis**	Uncommon	Common	Not specific
